# AI-Generated Versus Human Supervisor Feedback on Medical Students’ Clinical Clerkship Logs: Cross-Sectional Convergent Mixed Methods Study

**DOI:** 10.2196/90064

**Published:** 2026-06-16

**Authors:** Takeshi Kondo, Jeroen Donkers, Hiroshi Nishigori, Sanne Rovers, Sylvia Heeneman

**Affiliations:** 1 Center for Medical Education, Nagoya University Graduate School of Medicine, 65, Tsurumai-cho, Showa-ku, Nagoya, 466-8550, Japan, 81 52 741 2111; 2 School of Health Professions Education, Maastricht University, Maastricht, The Netherlands; 3 Department of Educational Research and Educational Design, Maastricht University, Maastricht, The Netherlands; 4 Department of Pathology, Maastricht University, Maastricht, The Netherlands

**Keywords:** artificial intelligence, clinical clerkship, feedback, large language model, medical education, mixed methods research

## Abstract

**Background:**

Feedback is essential for medical students’ learning during clinical clerkships; yet, supervising physicians often struggle to provide meaningful written feedback due to time constraints. Large language models offer a promising approach to supplement human feedback, but how artificial intelligence (AI)–generated and human feedback differ in authentic clinical settings remains unclear, as most comparisons have been conducted in classroom or simulation contexts.

**Objective:**

The aim of the study is to examine how AI-generated feedback and supervisor-provided feedback differ when applied to medical students’ clinical clerkship logs, by identifying the distinct characteristics and complementary strengths of each feedback type.

**Methods:**

This cross-sectional convergent mixed methods study included 161 weekly clinical clerkship logs from 47 fifth- and sixth-year medical students across 12 clinical departments at Nagoya University, Japan (January-May 2024). Of 164 eligible logs, 3 were excluded because supervisors entered contact messages rather than substantive feedback. AI feedback was generated using GPT-4o. In total, 10 faculty physicians and 10 medical students evaluated both feedback types in blinded, randomized order using a validated 5-category rubric (criteria-based, clear direction, accuracy, prioritization, and supportive tone), followed by open-ended comments and source identification. Quantitative analyses (paired 2-tailed *t* tests, cumulative link mixed-effects models; α=.05 with Bonferroni correction) were complemented by qualitative thematic analysis and integrated using joint display analysis.

**Results:**

AI feedback was significantly longer than supervisor feedback (mean 382.02, SD 81.82 vs mean 98.87, SD 73.66 characters; Cohen *d*=2.84, 95% CI 2.50‐3.19; *P*<.001). Cumulative link mixed-effects models showed that AI scored higher on criteria-based (odds ratio [OR] 11.81, 95% CI 7.64‐18.27; *P*<.001) and clear direction (OR 6.61, 95% CI 4.35‐10.06; *P*<.001), with no significant differences on accuracy (OR 1.35, 95% CI 0.91‐2.00; *P*>.99), prioritization (OR 1.70, 95% CI 1.16‐2.50; *P*=.10), or supportive tone (OR 1.34, 95% CI 0.87‐2.06; *P*>.99). AI feedback showed greater consistency (variance ratio 3.9:1; Levene *F*_1,320_=73.20; *P*<.001). All 20 evaluators correctly identified feedback sources. Qualitative analysis revealed that AI provided structured, text-anchored feedback addressing rubric criteria, while supervisors offered experience-based feedback grounded in clinical context and professional expertise.

**Conclusions:**

This study extends the comparison of AI-generated and supervisor feedback to an authentic clinical clerkship environment, moving beyond classroom and simulation settings examined in prior work. Through integrated mixed methods analysis, a key distinction emerged between text-anchored AI feedback, which systematically addresses written log content in alignment with rubric criteria, and experience-based supervisor feedback, which draws on clinical observation and professional judgment. AI consistently delivered structured feedback addressing gaps that arise when time-pressured supervisors provide brief comments, while supervisors contributed clinically grounded insights that AI cannot replicate. These complementary strengths suggest that AI feedback should supplement rather than replace supervisor feedback, and that hybrid models leveraging each type’s advantages warrant investigation in clinical education.

## Introduction

### Background

Feedback serves as a critical educational instrument for medical students and residents to learn from their experiences in the clinical environment [1-4]. Such feedback can be provided orally or as written feedback [5]. In the clinical environment, where multiple supervising physicians often take turns in providing guidance, obtaining and documenting written feedback is important for ensuring the continuity of learning and assessment [6-8]. However, supervising physicians in the clinical setting often have multiple tasks, which puts a strain on the time they have to support learning through feedback [9]. Particularly regarding written feedback, supervising physicians are often unable to provide this in a sufficiently elaborated manner [9,10].

To supplement feedback from human supervisors, feedback generation using artificial intelligence (AI) has recently gained attention. In particular, since the advent of large language models (LLMs), the ease of using AI has led to an enthusiastic exploration of its application in feedback [11-13]. LLMs are AI models that can handle various tasks by pretraining on massive amounts of text data in advance [14]. Although previous AI technologies were also able to generate feedback, they required models trained on context-specific text data tailored to the feedback’s domain [15]. In contrast, LLMs can adapt to diverse contexts with minimal modifications, making them promising tools for the generation of feedback [16,17]. When AI feedback and human feedback were compared for feedback generation for texts written by learners, it was shown that AI feedback can match that of well-trained educators in terms of appropriateness, usefulness of the content, and supportive tone [13,18,19]. However, even studies demonstrating that AI can generate high-quality feedback argue that AI feedback should complement rather than replace human feedback, citing student preferences and the responsibility that human instructors should maintain [20-22].

### Rationale and Research Question

When leveraging AI feedback to complement human feedback, it is important to understand how the 2 differ. Prior research has reported inconsistent findings regarding these differences. Several studies report that AI feedback is longer and more detailed than human feedback [20,23,24], whereas another study found AI feedback to be shorter and less relevant [25]. Although longer feedback is often associated with higher perceived quality [26-28], the extent to which differences in length between AI and human feedback translate into quality advantages remains unclear [18,20]. For example, in dental histology assignments, AI feedback was longer and contained more improvement points than human feedback [20]; yet, an analytical, multicriterion comparison showed that human teachers produced feedback that was overall clearer, more accurate, and better prioritized, even when shorter [18]. Regarding the consistency of feedback quality, many studies report that the quality of human feedback varies widely depending on the provider and the situation [29-31], whereas some studies find that, compared with AI, human feedback is consistently higher in quality [25,32]. Findings are also inconsistent about whether recipients can tell if the feedback provider is AI or human, ranging from studies reporting that people could distinguish it reasonably well [33] to studies reporting that they could hardly distinguish it [34,35]. Thus, although many aspects of AI and human feedback have been examined, the results vary widely. These divergences likely relate to contextual variation such as differences in discipline, task authenticity, and assessment purpose.

When comparing AI and humans in other clinical environments including clinical clerkships, a key limitation is that most existing comparisons have examined classroom or simulation tasks rather than authentic clinical activities [18,20,23,24]. In clinical clerkships, supervising physicians work closely with students. Their feedback can therefore draw on direct observation of student behavior, tacit professional norms, and shared patient-care experiences. These dimensions are difficult for AI to approximate, because AI has access only to the written log. Furthermore, prior comparative studies often allowed humans ample preparation time for crafting feedback [13,18,19], whereas in real clinical settings, supervisors are time-pressured and may produce brief or nonspecific comments [9,10]. This specific context constraint could both expose areas where human feedback quality diminishes and highlight domains where AI systems might compensate, given their ability to generate structured output rapidly.

Accordingly, an analytical examination situated within the clinical environment is needed to clarify how AI and human feedback differ when applied to authentic clerkship learning logs under routine time constraints. By exploring these differences, we can identify ways to leverage AI-generated feedback to complement supervisors in the busy clinical environment. Accordingly, our research question is: In what ways and along which dimensions do feedback from supervising physicians and feedback generated by AI differ when applied to medical students’ clinical clerkship logs?

## Methods

### Setting

The research was conducted within the clinical clerkship programs for fifth- and sixth-year medical students at Nagoya University (NU), Japan. In Japan, students enter medical school directly after high school (around age 18 years) and complete a 6-year program [36]. At NU, medical students finish classroom-based learning in clinical medicine and preparatory training for clinical clerkships (such as medical interviewing and physical examination) by the end of the fourth year. From late fourth to early fifth year, they undertake observation-centered clinical clerkships, rotating through all departments for short periods of 1‐2 weeks. From the late fifth to the early sixth year, they participate in clinically active clerkships, rotating through each department for 4 weeks or longer. For this study, we focused on the clinical clerkship program at NU for students in the latter half of their fifth year and throughout their sixth year. The participants were a single cohort of students who began their clinically active clerkships during their fifth year and continued into their sixth year as part of their normal academic progression (the Japanese academic year begins in April). During the clerkship, students joined the medical team, examined patients, formulated assessments and plans, and documented these in the electronic medical record. Because access to electronic medical records was rigidly restricted, students also recorded their daily activities, learning points, and future tasks in an e-portfolio system separate from the medical record. These clinical clerkship logs in the e-portfolio were used solely for educational purposes, and students were instructed not to include any patient-identifying information. Each daily log entry consisted entirely of free-text fields in which students described what they did during the clerkship, the cases they experienced, what they learned, and what they planned to do next; no Likert scales or structured questions were included, and no upper or lower character limits were imposed. Students entered their logs via smartphone. The e-portfolio automatically compiled the daily entries in the clerkship log into weekly records and sent them to the supervising physicians, who then provided written feedback to the students via the e-portfolio. Supervising physicians received orientation on the e-portfolio system but no specific training or instructions regarding the content, length, structure, or format of their written feedback. Written feedback in the e-portfolio primarily served as a complement to verbal feedback provided during clinical supervision, although the relative emphasis varied across supervisors and departments.

### Design

**Figure 1.**
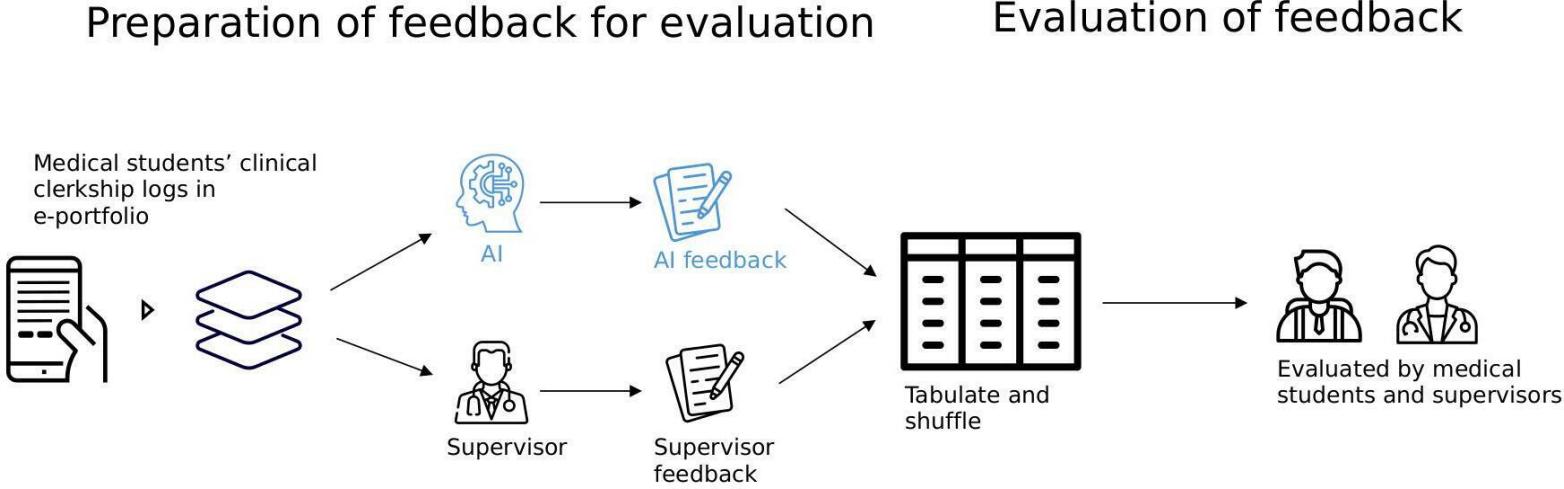
Overview of the feedback preparation and evaluation process in a mixed methods study comparing AI-generated and supervisor feedback on clinical clerkship logs at Nagoya University, Japan. Student logs from the e-portfolio were used to generate AI feedback (GPT-4o) and collect supervisor feedback, which were then tabulated, shuffled, and evaluated by blinded medical students and faculty evaluators. AI: artificial intelligence.

This study was conducted as a cross-sectional mixed methods study using a convergent design from a pragmatic perspective [37,38]. From the pragmatist standpoint of gaining insights that contribute to building a system where AI and supervisors complement each other to promote student learning, we examine the differences between AI-generated feedback and human-written feedback on clinical clerkship logs using both quantitative and qualitative approaches. First, we collected feedback from supervising physicians on clinical clerkship logs entered by medical students in the e-portfolio (supervisor feedback) and generated feedback using AI on the clinical clerkship logs (AI feedback), see Figure 1 for further details of this preparation process. Next, within each dataset, the presentation order of the 2 feedback types (AI vs supervisor) was randomly assigned so that approximately half of the datasets presented AI feedback first and the other half presented supervisor feedback first. This randomization was intended to prevent evaluators from deducing the source of each feedback from its position. The 161 datasets were then randomly distributed among the 10 faculty evaluators and 10 student evaluators. Because the assignment was random, some evaluators assessed multiple records originating from the same clinical department. Then, medical students and supervising physician evaluators were asked to evaluate both types of feedback. The evaluation used the rubric developed by Steiss et al [18] for quantitative analysis of scores and qualitative analysis of free-text comments. Finally, the results of the quantitative and qualitative analyses were integrated using joint display analysis (JDA) [39,40], in which each quantitative finding was placed alongside its corresponding qualitative theme in a structured display, and meta-inferences about how the 2 sources complement or diverge from each other were drawn. The detailed integration procedure is described later in the Integration of Results section. The process of preparing and evaluating AI feedback and supervisor feedback is shown in Figure 1.

### Participants

#### Inclusion and Exclusion

During the data collection period (January 9 to May 5, 2024), 164 weekly clerkship logs from 47 fifth- and sixth-year medical students across 12 clinical departments (9 university departments and 3 community hospitals) were assessed for eligibility. In total, 3 logs were excluded because the supervising physician had entered contact messages to students rather than substantive feedback, yielding 161 logs for analysis.

For the evaluation phase, eligibility criteria for faculty evaluators included current involvement in teaching medical students; for student evaluators, eligibility required current participation in clinical clerkships or preparatory education for clinical clerkships. Individuals who authored the student logs used as the basis for feedback, as well as those who authored the supervisor feedback being evaluated, were excluded.

#### Participant Characteristics

The study involved 2 participant groups: log authors (47 medical students from a single cohort transitioning from fifth to sixth year during the study period) and feedback evaluators (10 faculty physicians and 10 medical students). Detailed demographic information (age and sex) was not collected from either group; because evaluators participated as unpaid volunteers, the information requested was kept to a minimum to reduce burden and invasiveness.

#### Sampling Procedures

Student clerkship logs were collected as part of routine educational practice through the e-portfolio system; all logs from the study period were included unless they failed the eligibility criterion described earlier. Faculty evaluators were recruited via a mailing list for medical educators, and student evaluators were recruited through student representatives who disseminated information about the study via social media. All evaluators received an explanation about the significance and purpose of the study from the first author (TK) through documentation and an online meeting prior to their participation.

### Materials

#### Generating AI Feedback

On the same set of weekly records of a medical student, feedback was also generated by GPT-4o (gpt-4o-2024-05-13) from OpenAI, which was the latest AI model available from OpenAI as of June 1, 2024, when this feedback was generated. This feedback is called “AI feedback” below.

#### Prompt Development

To generate high-quality feedback through LLMs, it is crucial to carefully craft the prompts provided to the model [41]. Since the students’ clinical clerkship logs are written in Japanese and the feedback is for Japanese students, we created the prompts in Japanese. Although Steiss et al [18] dealt with different types of feedback, their study also compared human-generated descriptions with feedback produced by ChatGPT in response to written tasks. Given the similarities, we decided to adapt the prompts from Steiss et al [18] as a foundation for our current research. TK, who is a Japanese speaker and familiar with the context where feedback is applied, translated them into Japanese. Additionally, TK referenced papers on prompt creation methods for high-quality feedback generation, adding context and mission elements, as well as criteria for high-quality feedback [41]. We then fed these refined prompts and actual student experiential logs into ChatGPT (GPT-4o) web interface to generate feedback. Next, we compared the generated feedback against previously reported quality criteria [18,42] and fine-tuned the prompts accordingly. Finally, to enhance reproducibility and subsequent integration into our e-portfolio system, we used the corresponding application programming interface (API) of the ChatGPT model (OpenAI Chat Completion API [gpt-4o-2024-05-13]) [43]. Each student log was processed as an independent API call with no shared context between calls, ensuring that feedback for one log could not be influenced by any other log. No maximum token limit (max_tokens parameter) was set at the API level.

The prompt was structured to include sections that established the premise of providing feedback on clinical clerkship logs, explained the structure of these logs, outlined the evaluation criteria for the feedback, and specified the expected content and format of the output. The prompt included an explicit instruction to write the feedback concisely within 500 characters. This character limit was determined through systematic pilot testing, in which feedback was generated under various length constraints (no limit, 200, 300, 400, and 600 characters); without any limit, the AI produced verbose output, in which key points were diluted, whereas overly restrictive limits resulted in superficial feedback. The 500-character limit was selected as the optimal balance between conciseness and substantive coverage. Supervising physicians received no instructions regarding length, structure, or format for their feedback. For an outline of the prompt construction and revision process, we refer to Multimedia Appendix 1. Since the logs were written in Japanese, the prompt was also developed in Japanese. A translation in English of the final prompt is included in the appendix as well as the original Japanese version.

An important methodological note regarding this design: the prompt explicitly embedded the following 5 criteria from the Steiss et al [18] rubric as the evaluation framework for generating feedback: criteria-based guidance, clear directions for improvement, accuracy, appropriate prioritization, and supportive tone. The AI was instructed to address each criterion in its output. Supervising physicians, by contrast, received no instructions to write in accordance with this rubric and provided feedback according to their usual clinical practice. This asymmetry means that the study compares a rubric-optimized AI system with naturalistic supervisor feedback rather than constituting a neutral comparison of inherent feedback quality.

#### Evaluation Instrument

The evaluation instrument consisted of closed-ended and open-ended questions. For the closed-ended questions, we used the scoring rubric developed by Steiss et al [18]. While there are other criteria used in studies comparing human and AI feedback, most of them used holistic scoring methods [13,19]. In contrast, Steiss et al [18] used analytical criteria that evaluate feedback by breaking it down into various elements. This approach aligns with our objective of analyzing how different aspects of feedback differ between humans and AI. This rubric evaluates 5 categories: criteria-based, clear directions for improvement, accuracy, prioritization of essential features, and supportive tone, each rated with a single score on a 5-point scale. A score of 5 represents the best evaluation, while 1 represents the worst. Each category has descriptive criteria attached. The rubric is presented in Multimedia Appendix 2. Because this rubric emphasizes structural dimensions of feedback quality rather than clinically specific qualities such as diagnostic reasoning and professional judgment, a complementary qualitative component was incorporated to capture dimensions of feedback value that quantitative rubric scoring alone cannot assess. The methodological implications of the alignment between the AI prompt and this rubric for interpreting score comparisons are discussed in the Prompt Development section and the Limitations section.

The feedback in this study is in Japanese, and the evaluators are also Japanese, so it was necessary to translate the scoring rubric from English to Japanese. The translation process was conducted based on the translation, review, adjudication, pretesting, and documentation model [44]. This model is designed for the translation of evaluation forms, aiming to perform accurate translations while adapting to linguistic characteristics and cultural contexts that are difficult to address in traditional translation processes and to maintain the intent of the evaluation forms. This model follows five steps: (1) translation, (2) review, (3) adjudication, (4) pretesting, and (5) documentation. Following these steps, the translation was carried out as described below.

The original feedback quality evaluation rubric [18] was adapted for this study by modifying context-specific terms to fit the current research on student logs. The translation was performed by 2 bilingual individuals (TK and HN) who are fluent in both Japanese and English and experienced in medical education. The translations were reviewed by a medical education expert. Discrepancies were resolved by a professional translator. Pretesting involved a clinical educator and a medical student, leading to minor adjustments while preserving the original intent. These detailed processes were documented (Multimedia Appendix 3). This process ensured that the translated questionnaire was culturally and contextually appropriate for use in Japan.

In addition to the rubric, the instrument included a closed question asking evaluators whether the feedback had been written by a human or generated by AI, as well as an open-ended section in which evaluators described their impressions of the feedback’s strengths and areas for improvement. The administration sequence of these components is described in the Data Collection section.

### Sample Size, Power, and Precision

The required sample size for the 2-tailed *t* test was calculated using the values published in the paper by Steiss et al [18] that developed the rubric used for quantitative analysis. The paper reported effect sizes in terms of partial eta-square and Cohen *d*, which were used to calculate the sample size. In this study, considering the comparison between AI and supervisor feedback, the sample size was calculated based on Cohen *d* of 0.34 as reported by Steiss et al [18]. With power set at 0.8 and α at .05, a total of 155 data points per group would be needed.

### Data Collection

The 161 datasets were evaluated between October 2024 and January 2025. For each feedback entry, evaluators first completed the 5-item rubric scoring and then provided free-text comments describing their impressions of the feedback’s strengths and areas for improvement. Only after completing all rubric scoring and free-text comments was the identification question presented, asking evaluators to judge whether the feedback had been written by a human or generated by AI. This sequence was designed so that knowledge or suspicion of the source could not influence quality ratings, given that previous research has shown the feedback provider can influence how the feedback is perceived [45,46]. Whether the feedback had been generated by AI or written by a supervising physician was not disclosed to evaluators at the time of evaluation, ensuring they based their assessments solely on the feedback content.

### Quantitative Analysis

All quantitative analyses were conducted in R (version 4.4.2; R Foundation for Statistical Computing). Text length was operationalized as the number of Unicode characters (Unicode Transformation Format-8) in each feedback entry. Since Japanese writing does not use spaces between words, and word counts can vary depending on morphological analysis methods, we used character counts as a more stable metric rather than word counts. Although it varies by domain, Japanese is reported to have approximately 1.7 characters per word [47]. Because each student record had both an AI and a supervisor feedback instance, analyses were paired at the record level, and all tests were 2-sided with α=.05; 95% CIs are reported. To examine whether AI and supervisor feedback differed in length, we compared character counts using a paired Student *t* test across records and visualized the distributions to contextualize any difference in means. To compare perceived quality, we analyzed rubric scores (5 ordinal categories: criteria-based, clear direction, accurate, prioritization, and supportive) in 2 complementary ways. First, for an easily interpretable summary at the record level, we averaged available assessor scores (student and faculty) within provider and item and then used paired 2-tailed *t* tests to compare AI and supervisor mean scores across records. Second, acknowledging the ordinal nature of the outcomes, we fitted cumulative link mixed-effects models. For each item, the fixed effects were feedback provider (AI vs supervisor) and assessor type (student vs faculty), with their interaction examined exploratorily, and a random intercept for assessor to account for clustering. Results are presented as odds ratios (ORs) with 95% CIs and *P* values under the proportional-odds assumption. To assess consistency in feedback quality, we computed the per-record mean score for each provider and compared dispersion between AI and supervisors. Variance differences were tested using the Levene test for equality of variances and corroborated with the classical *F* test, alongside distributional plots of the per-record means. To evaluate whether longer feedback tended to receive higher scores, we estimated Spearman rank correlations between character count and rubric scores separately by provider and item, reporting correlation coefficients and 2-sided *P* values. Finally, for the identification task, we summarized detection accuracy as the proportion of correctly identified sources (AI vs supervisor). Bonferroni correction was applied to correct for multiple testing.

### Qualitative Analysis

#### Overview

For the qualitative analysis, narrative comments from the open questions were organized in a table, followed by a thematic analysis [48]. Thematic analysis consists of the following steps: (1) familiarize with the data, (2) generate initial codes, (3) search for themes, (4) review themes, (5) define and name themes, and (6) produce the report [48]. Codes were generated inductively from the evaluators’ free-text comments rather than being mapped onto a predefined coding framework. Steps 1 and 2 were conducted by 2 individuals (TK and HN). TK created the initial codes, while HN reviewed and refined them. Coding meetings between TK and HN were held in person, and theme-development meetings with SH, SR, and JD (step 3 onward) were conducted via videoconference. When coding or theme-level disagreements arose, the analysts returned to the original data (Japanese excerpts at the initial coding stage and English-translated codes during theme development), examined alternative interpretations, and continued discussion until consensus was reached before advancing to the next step. Steps 1 and 2 were conducted in Japanese, while steps 3 to 6 were conducted in English. From step 3 onward, the codes were translated into English, and SH, SR, and JD also participated. This allowed for a more multifaceted analysis. Codes and themes were organized in Google Sheets and Microsoft Excel; R (version 4.4.2; the same version used for the quantitative analyses) was used for tabulating the code list and computing simple frequency summaries. No dedicated computer-assisted qualitative data analysis software (CAQDAS) was used. The backgrounds of TK, HN, SH, SR, and JD are described below.

#### Reflexivity

TK is a general practitioner directly involved in clinical clerkships and also engaged in the development of e-portfolios and feedback. HN is an expert in medical education, researching professionalism in medical education. SH researches e-portfolios and programmatic assessment in health professions education, SR is an expert in self-regulated learning and assessment, and JD is a specialist in statistics and AI.

TK’s knowledge about the context of clinical clerkship feedback provision helps in making contextually informed deeper analysis when interpreting descriptions, while TK’s involvement in the development of e-portfolios might lead to potential bias where results could be interpreted more favorably toward e-portfolios. The specialized fields of HN, SH, SR, and JD each provide expert perspectives when analyzing descriptions. On the other hand, there is a possibility of biased interpretations based on their respective specialties. To mitigate such biases, discussions were recorded, and transparency was maintained during the analysis process, with regular meetings to share and discuss their respective interpretations. The research team had no direct supervisory or assessment-related authority over the evaluators who provided the qualitative data; evaluators were recruited independently (see Sampling Procedures section) and participated on an opt-in voluntary basis.

#### Methodological Integrity

Methodological integrity was pursued along the 2 dimensions articulated in the APA Journal Article Reporting Standards for Qualitative Research [49]: fidelity to the subject matter and utility in achieving the research goals. With respect to data adequacy, the analytic corpus comprised the complete set of open-ended comments from all 20 evaluators (10 faculty and 10 students) across 161 datasets spanning 12 clinical departments; because this full-corpus approach precluded iterative sampling to saturation in the conventional sense, we treated the breadth of the evaluator pool (2 assessor types and multiple specialties) as the operational criterion for sufficiency. Themes are illustrated in the Results section with verbatim excerpts to maintain groundedness.

Credibility was supported by several analytic checks. Initial coding (steps 1 and 2) was double-coded by TK and HN, with discussion to agreement. Theme development (steps 3 to 6) was opened to 3 additional analysts (SH, SR, and JD) with distinct disciplinary perspectives, functioning as analyst triangulation across clinical practice, medical education, programmatic assessment, self-regulated learning, and statistics and AI. The bilingual workflow (Japanese for initial coding and English for theme development) further served as a translational check on interpretive fidelity. All analytic discussions were recorded, providing an audit trail. Themes were iteratively examined for within-theme disconfirming instances, and cases contradicting the dominant pattern (eg, brief supervisor feedback that was nonetheless judged effective or AI feedback judged overly prescriptive) were retained in the theme descriptions rather than treated as outliers.

Member checking was not conducted because evaluators participated as unpaid volunteers, including sixth-year students approaching the national licensing examination and clinically active faculty, for whom additional engagement would have been incompatible with our commitment to minimize participant burden (see Ethical Considerations section). Credibility was instead supported by the multianalyst procedures described earlier. Meaningful coherence between findings and interpretation was pursued through JDA, in which each quantitative result was aligned with its corresponding qualitative theme (see Integration of Results section). To support transferability, the Setting section provides detailed contextual information about the Japanese undergraduate clinical clerkship environment so readers can judge applicability to other educational contexts.

### Integration of Results

The results of the quantitative and qualitative analyses were documented separately and then integrated using JDA. JDA is an analytic strategy in mixed methods research that brings qualitative and quantitative evidence together in a single, structured visual (a “joint display”) to enable explicit integration and the drawing of meta-inferences that would be difficult to see from either strand alone [39,40]. In this study, the results of the quantitative and qualitative analyses were compared and integrated using JDA. Specifically, the quantitative results were tabulated, and the themes identified in the qualitative analysis were listed alongside them. The relationships between the quantitative results and qualitative themes were then examined, and meta-inferences were drawn.

### Ethical Considerations

This study was approved by the ethics committee of Nagoya University Hospital (approval: 2023‐0451). An opt-out approach was taken for the research use of student clinical clerkship logs and supervisor feedback accumulated in the e-portfolio. Before the clerkship, all medical students received an explanation from TK via online meetings regarding the use of clinical clerkship logs in e-portfolio for research purposes and were informed that they could opt out by sending an email to a designated address. Supervising participants received explanations from TK via email and, upon request, online meetings regarding the use of feedback recorded in the e-portfolio and were likewise informed that they could opt out by sending an email to a designated address. Research participants who evaluated AI feedback and supervisor feedback were recruited using an opt-in approach. All evaluators provided informed consent before participating. Student clinical clerkship logs, supervisor feedback, AI feedback, and evaluator assessment data used in the research were anonymized for analysis. All proper nouns were replaced with alphabetic characters, and linking tables were managed in an encrypted state. Evaluators received no financial compensation for their participation; as acknowledgment of their contribution, they were provided with information about the research and early access to the AI prompt used for feedback generation. No individually identifiable information of any participant is presented in this paper, tables, figures, or supplementary materials.

## Results

### Overview

During the clinical clerkship conducted at NU from January 9, 2024, to May 5, 2024, combinations of student clerkship logs and corresponding supervisor feedback were collected. The data included 9 clinical departments and 3 community hospitals, involving a total of 47 medical students and 42 supervisors who contributed to the clerkship logs and feedback. On June 1, 2024, AI feedback was generated for the student clerkship logs. As a result, a total of 161 datasets were created, each consisting of a student’s clerkship logs for 1 week, supervisor feedback, and AI feedback. The weekly clerkship logs varied considerably in length (mean 675.2, SD 459.8; range 51‐2342; median 551, IQR 313-1021 characters). The number of datasets, students, and supervisors by department is shown in Table 1.

**Table 1.** Dataset summary^a^.

Department^b^	Datasets, n	Students, n^c^	Supervisors, n^d^
A Community Hospital	13	11	10
B Community Hospital	7	6	7
C Community Hospital	4	4	3
Diabetes and Endocrinology	9	8	9
General Medicine	5	5	5
Geriatric Medicine	16	12	10
Hematology	11	10	10
Neurology	16	12	15
Pediatrics	32	22	19
Psychiatry	9	7	8
Surgical Unit	23	18	17
Thoracic Surgery Unit	16	12	14

a Convergent mixed methods study comparing artificial intelligence–generated and supervisor feedback on clinical clerkship logs, Nagoya University, Japan, 2024.

b Except for A, B, and C Community Hospital, all departments are affiliated with Nagoya University.

c The total number of students exceeds 47 because some students rotated through multiple departments.

d The total number of supervisors exceeds 42 because some supervisors provided feedback in multiple departments.

**Figure 2.**
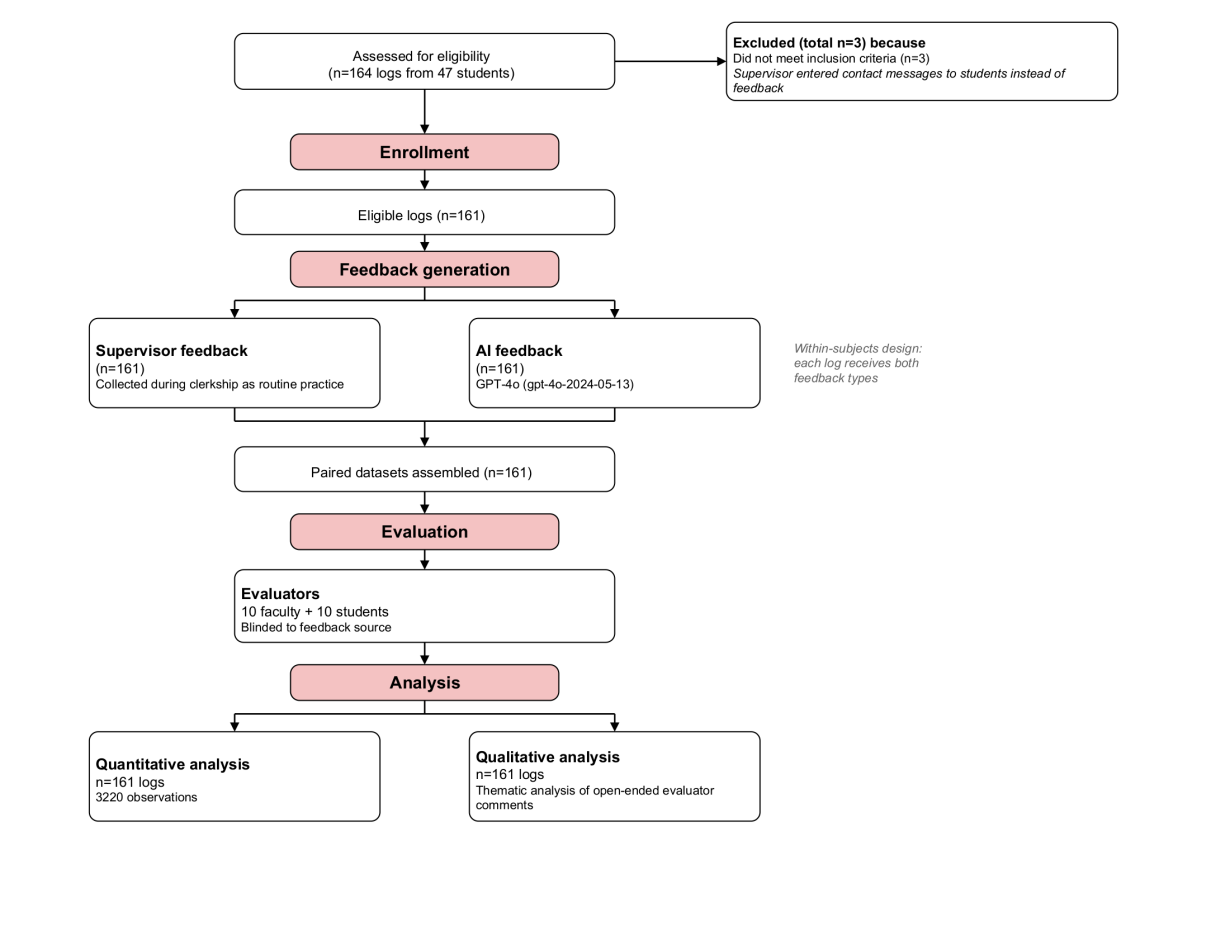
Participant flowchart for a mixed methods study comparing AI-generated and supervisor feedback on clinical clerkship logs at Nagoya University, Japan (January 2024-January 2025). The diagram shows participant flow from enrollment (164 logs from 47 medical students across 12 departments) through exclusion (n=3), feedback generation (within-subjects design: each log received both AI and supervisor feedback), blinded evaluation by 10 faculty and 10 student evaluators, to quantitative (3220 observations) and qualitative analyses. AI: artificial intelligence.

From October 2024 to January 2025, 10 medical students and 10 faculty who were not involved in the creation of the above student clerkship logs or feedback evaluated the AI and supervisor feedback. No missing data were identified; all 161 records contained complete rubric scores from both assessors across all 5 items, yielding 3220 observations with 100% data completeness. Because the dataset was complete, we did not perform a missingness test (such as Little missing completely at random test) or apply multiple imputation. The flow of participants through each stage of the study is shown in Figure 2.

### Quantitative Analysis

#### Distribution Assessment and Sensitivity Analysis

Shapiro-Wilk tests assessed the normality of paired differences for all comparisons. The feedback length difference was normally distributed (*W*=0.992; *P*=.55), supporting the use of the paired 2-tailed *t* test for that variable. However, all 5 rubric score differences departed significantly from normality (all *W*≤0.939; all *P*<.001 after Bonferroni correction). As a sensitivity analysis, Wilcoxon signed rank tests were conducted alongside the paired 2-tailed *t* tests; the 2 methods yielded identical conclusions regarding statistical significance for every variable. Moreover, the primary analysis of rubric scores used cumulative link mixed-effects models (see the Comparison of Scores section), which do not assume normality of the outcome distribution.

#### Difference Between Feedback Length

**Figure 3.**
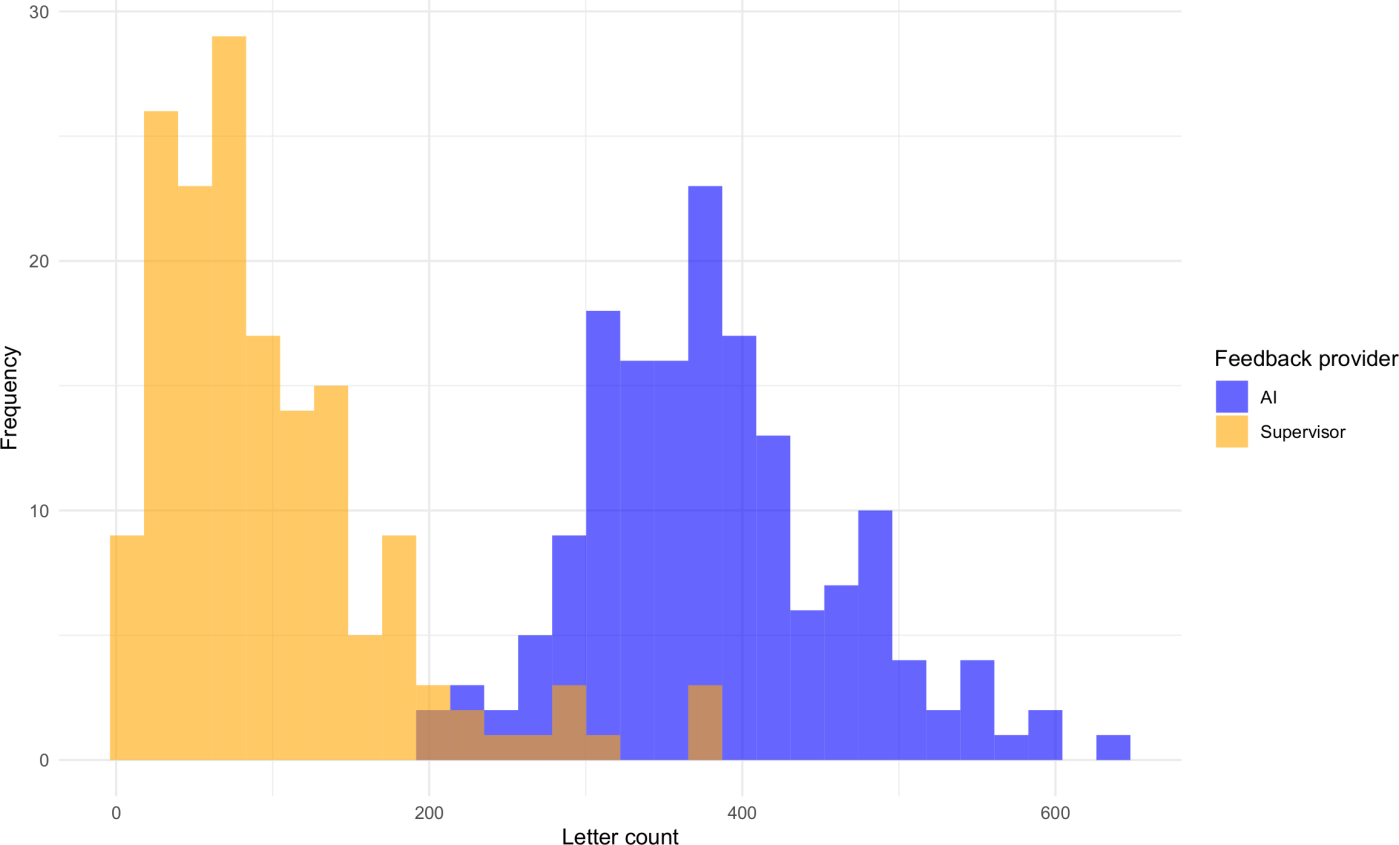
Distribution of AI-generated and supervisor feedback lengths by character count for 161 clinical clerkship logs from 47 medical students, Nagoya University, Japan, 2024. AI: artificial intelligence.

The length of the feedback was compared between AI and supervisor feedback. Mean lengths were 382.02 (95% CI 369.29‐394.76) characters for AI and 97.87 (95% CI 86.4‐109.33) characters for supervisors. The distribution of the lengths of supervisor feedback and AI feedback is shown in Figure 3. Paired 2-tailed *t* test results indicated that the mean character count of AI feedback was significantly higher than that of supervisor feedback (mean difference 284.16, 95% CI 268.6‐299.71 characters; *P*<.001; Cohen *d*=2.84, 95% CI 2.50‐3.19).

#### Comparison of Scores

The scores assigned by the evaluators to the AI and supervisor feedback were compared. Multimedia Appendix 4 shows the differences in feedback scores by AI and supervisors for the same log. Values greater than 0 indicate that the AI score is higher, and values less than 0 indicate that the supervisor score is higher. Although there are slight differences among evaluators, AI scores tend to be higher except for the “supportive” item.

Next, we compared the mean scores of AI and supervisor feedback for each category. In all categories, AI feedback had higher mean scores than supervisor feedback. A 2-tailed *t* test comparing the mean scores of supervisor feedback and AI feedback showed that AI feedback was rated significantly higher in all categories after Bonferroni correction: criteria-based (corrected *P*<.001), clear direction (corrected *P*<.001), prioritization (corrected *P*<.001), accurate (corrected *P*<.001), and supportive (corrected *P*=.01). Details are shown in Table 2; mean differences and uncorrected *P* values are provided in Multimedia Appendix 5.

**Table 2.** Paired *t* test results comparing artificial intelligence (AI) and supervisor feedback scores on the Steiss et al 5-item analytic rubric^a^.

Item	AI mean score (95% CI)	Supervisor mean score (95% CI)	Cohen *d* (95% CI)	Corrected *P* value
Criteria-based	4.43 (4.34‐4.52)	2.73 (2.56‐2.9)	0.93 (0.79-1.06)	<.001
Clear direction	4.36 (4.26‐4.46)	2.98 (2.81‐3.14)	0.73 (0.61-0.86)	<.001
Accurate	4.18 (4.07‐4.28)	3.8 (3.64‐3.96)	0.22 (0.11-0.33)	<.001
Prioritization	4.05 (3.94‐4.16)	3.37 (3.2‐3.54)	0.36 (0.24-0.47)	<.001
Supportive	4.56 (4.47‐4.65)	4.33 (4.21‐4.45)	0.17 (0.06-0.28)	.01

a Convergent mixed methods study, Nagoya University medical students, Japan, 2024. Rubric items: criteria-based, clear direction, accurate, prioritization, and supportive. This rubric assesses structural and content dimensions of written feedback; clinically specific dimensions such as diagnostic reasoning and professional judgment were not directly measured. Mean differences and uncorrected *P* values are reported in Multimedia Appendix 5.

Because the rubric used for scoring is a 5-point scale and the intervals from 1 to 5 may not be equal, using average scores may not be appropriate. Therefore, we used ordinal logistic regression to examine whether there are differences between AI and supervisor scores. We also used a mixed-effects ordinal model to account for assessor type (student or faculty) and random effects by assessor. The results are shown in Table 3. In the ordinal logistic regression, 3 effects are expressed as OR for each category. “Provider: AI” indicates that the larger the OR above 1, the more likely AI is to receive higher scores compared to supervisors. “Assessor type: student” indicates that the larger the OR above 1, the more likely students are to give higher scores compared to faculty. “Interaction: supervisor×student” indicates that the larger the OR above 1, the greater the difference in scores between AI and supervisors among students compared to faculty. *P* values are Bonferroni-corrected for comparisons across 15 items. The results indicated that, for the items criteria-based and clear direction, AI feedback received significantly higher scores than supervisor feedback (criteria-based: OR 11.81, 95% CI 7.64‐18.27; *P*<.001 and clear direction: OR 6.61, 95% CI 4.35‐10.06; *P*<.001; OR values larger than 1 indicate higher scores for AI than for supervisors). For accurate, prioritization, and supportive, AI also tended to receive higher scores, but the differences between AI and supervisors were not significant (accurate: OR 1.35, 95% CI 0.91‐2.00; *P*>.99; prioritization: OR 1.70, 95% CI 1.16‐2.50; *P*=.10; and supportive: OR 1.34, 95% CI 0.87‐2.06; *P*>.99). Regarding assessor type (student vs faculty), no significant differences were observed in any of the items. We also included interaction terms to test whether the effects of AI versus supervisor depended on assessor type, but no significant interaction effects were observed for any item.

**Table 3.** Cumulative link mixed-effects models results comparing artificial intelligence (AI) and supervisor feedback scores on the Steiss et al 5-item analytic rubric^a^.

Item and effect		OR^b^ (95% CI)	*P* value	Corrected *P* value
Criteria-based				
Provider: AI (vs supervisor)		11.81 (7.64‐18.27)	<.001	<.001
Assessor type: student (vs faculty)		1.60 (1.07‐2.39)	.02	.33
Interaction: supervisor×student		0.88 (0.49‐1.59)	.68	.99
Clear direction				
Provider: AI (vs supervisor)		6.61 (4.35‐10.06)	<.001	<.001
Assessor type: student (vs faculty)		1.37 (0.92‐2.04)	.12	.99
Interaction: supervisor×student		1.09 (0.61‐1.95)	.78	.99
Accurate				
Provider: AI (vs supervisor)		1.35 (0.91‐2.00)	.14	.99
Assessor type: student (vs faculty)		1.77 (1.16‐2.72)	.008	.13
Interaction: supervisor×student		0.98 (0.55‐1.76)	.96	.99
Prioritization				
Provider: AI (vs supervisor)		1.70 (1.16‐2.50)	.007	.10
Assessor type: student (vs faculty)		1.25 (0.83‐1.89)	.28	.99
Interaction: supervisor×student		1.62 (0.92‐2.86)	.10	.99
Supportive				
Provider: AI (vs supervisor)		1.34 (0.87‐2.06)	.19	.99
Assessor type: student (vs faculty)		1.57 (0.99‐2.49)	.06	.83
Interaction: supervisor×student		1.19 (0.61‐2.34)	.60	.99

a Convergent mixed methods study, Nagoya University medical students, Japan, 2024. See Table 2 for rubric item descriptions and measurement scope.

b OR: odds ratio.

These rubric-based findings should be interpreted in light of the asymmetric design described in the Methods section: the rubric criteria were explicitly embedded in the AI prompt but not given to supervisors. The observed score differences therefore reflect adherence to these specific structural dimensions rather than overall educational quality. The qualitative findings, particularly the themes of perspective as a clinician and continuity with practice, provide complementary evidence of the clinical value that supervisors contribute and that this rubric does not capture.

#### Consistency of Rubric Scores

**Figure 4.**
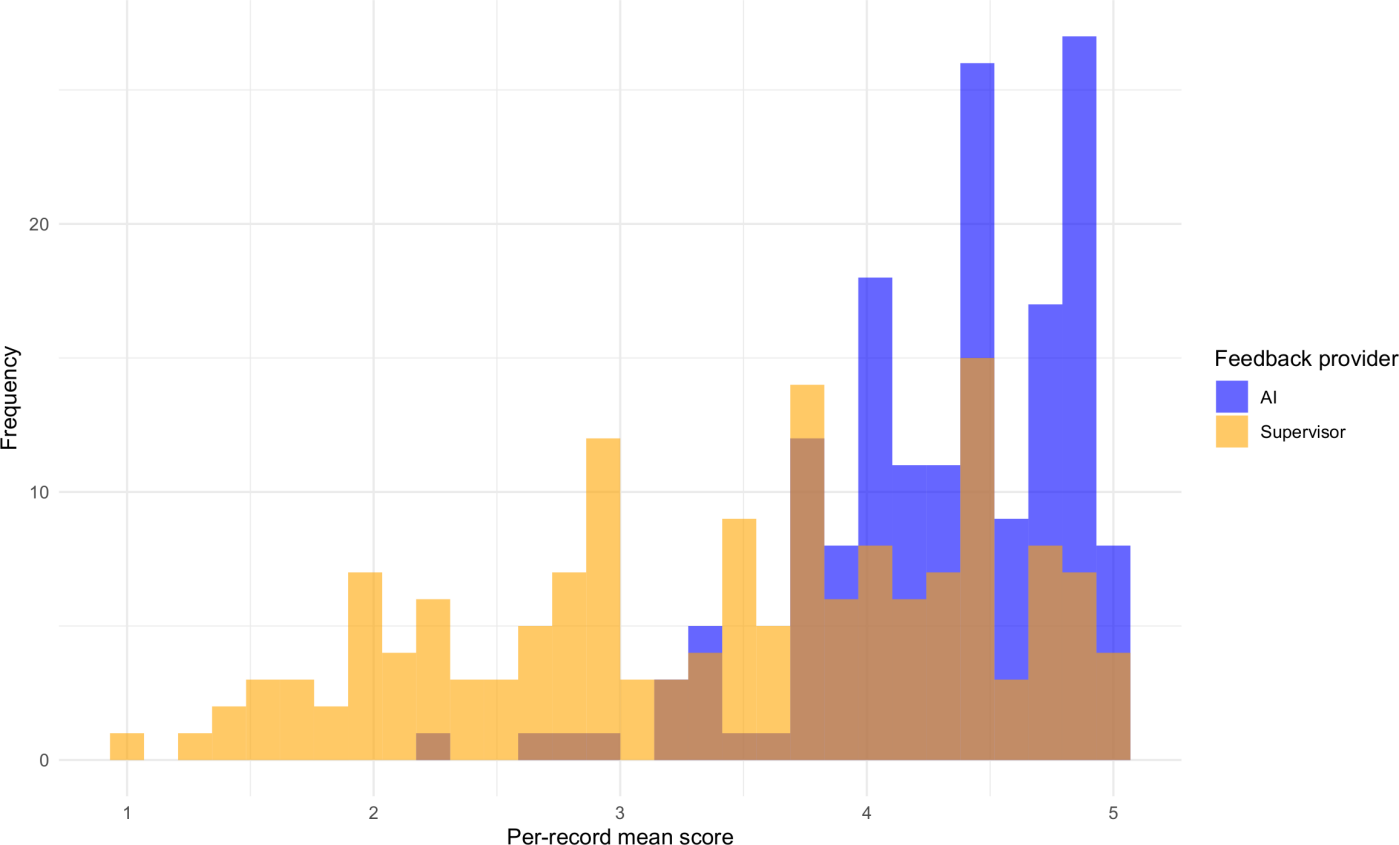
Distribution of per-record mean rubric scores for AI-generated and supervisor feedback on 161 clinical clerkship logs, Nagoya University, Japan, 2024. AI feedback shows smaller dispersion, indicating higher consistency. AI: artificial intelligence.

To compare the consistency of rubric scores between AI and supervisors, we plotted the distribution of per-clinical clerkship log mean scores (Multimedia Appendix 4Figure 4). AI exhibited smaller score dispersion, suggesting higher consistency in rubric scores. We further tested whether the variance of per-clinical clerkship log mean scores differs between AI and supervisors. The per-clinical clerkship log mean score was significantly more dispersed for supervisors (mean 3.44, SD 1.01; variance 1.011) than for AI (mean 4.32, SD 0.51; variance 0.262; Levene: *F*_1,320_=73.203; *P*<.001; *F* test: *F*_160,160_=0.259; *P*<.001). The supervisor variance was about 3.9 times that of AI, suggesting that AI feedback received more consistent rubric scores, whereas supervisor feedback received more variable ones.

#### Correlation Between Feedback Length and Score

To investigate whether the character count of feedback is correlated with the scores given by evaluators, we calculated the Spearman rank correlation coefficient for each feedback provider. The results are summarized in Table 4. *P* values are Bonferroni-corrected for comparisons across 10 items. A significant positive correlation would suggest that longer feedback tends to receive higher scores. No significant correlation was observed between text length and score for AI, but a significant correlation was found for all item categories for supervisors. This suggests that longer feedback from supervisors tends to receive higher scores.

**Table 4.** Spearman rank correlation between feedback character count and rubric score by feedback provider^a^.

Item	Spearman ρ		Corrected *P* value	
	AI^b^	Supervisor	AI	Supervisor
Accurate	0.04	0.50	>.99	<.001
Clear direction	0.00	0.63	>.99	<.001
Criteria-based	0.08	0.60	>.99	<.001
Prioritization	−0.02	0.58	>.99	<.001
Supportive	0.00	0.30	>.99	<.001

a Convergent mixed methods study, Nagoya University medical students, Japan, 2024. *P* values are Bonferroni-corrected for 10 comparisons.

b AI: artificial intelligence.

#### Relationship Between Student Log Length and Feedback Scores

Because AI feedback is tightly text-anchored, variation in student log quality could potentially influence feedback scores. To examine whether log length influenced rubric scores, Spearman correlations were calculated between weekly log character count and each rubric score, separately by feedback provider. After Bonferroni correction for 10 comparisons, no significant correlations were observed for either AI or supervisor feedback (all corrected *P*>.05), indicating that variation in student log length did not systematically influence rubric scores for either feedback type.

#### Interrater Reliability

Because each dataset was independently evaluated by 1 faculty member and 1 student, we examined agreement between these 2 assessor types using weighted Cohen κ (quadratic weights) and intraclass correlation coefficients, computed separately for AI and supervisor feedback across all 5 rubric items (Table 5). For supervisor feedback, interrater agreement was fair to moderate (weighted κ=0.37‐0.54; all *P*<.001). For AI feedback, agreement was poor (weighted κ=0.04‐0.15, mostly nonsignificant). This pattern suggests that faculty and student evaluators reached more consistent judgments when rating supervisor feedback than when rating AI feedback.

**Table 5.** Interrater reliability between faculty and student evaluators for artificial intelligence (AI)–generated and supervisor feedback^a^.

Item and feedback type	Values, n	% Agreement	Weighted κ	κ, *P* value	ICC^b^ (95% CI)	ICC, *P* value
Accurate						
AI	161	36	0.062	.41	0.062 (−0.087 to 0.210)	.21
Supervisor	161	41	0.383	<.001	0.384 (0.246 to 0.508)	<.001
Clear direction						
AI	161	44.7	0.150	.047	0.151 (−0.004 to 0.298)	.03
Supervisor	161	37.9	0.535	<.001	0.536 (0.417 to 0.638)	<.001
Criteria-based						
AI	161	44.1	0.076	.31	0.076 (−0.080 to 0.229)	.17
Supervisor	161	31.1	0.427	<.001	0.428 (0.293 to 0.547)	<.001
Prioritization						
AI	161	24.2	0.042	.57	0.042 (−0.107 to 0.191)	.29
Supervisor	161	32.9	0.379	<.001	0.381 (0.241 to 0.505)	<.001
Supportive						
AI	161	49.7	0.073	.30	0.073 (−0.083 to 0.226)	.18
Supervisor	161	55.9	0.365	<.001	0.367 (0.226 to 0.492)	<.001

a Convergent mixed methods study, Nagoya University, Japan, 2024. Weighted Cohen κ (quadratic weights) and ICCs were computed for each rubric item.

b ICC: intraclass correlation coefficient.

#### Identification of Feedback Source

After completing all rubric scoring and free-text comments, evaluators were asked whether they could identify the source of the feedback, specifically whether it was generated by a human or an AI. Despite the blinding procedures (randomized presentation order and removal of source labels), all students and supervisors correctly identified the source for every item (100% detection accuracy).

### Qualitative Analysis

#### Overview

**Figure 5.**
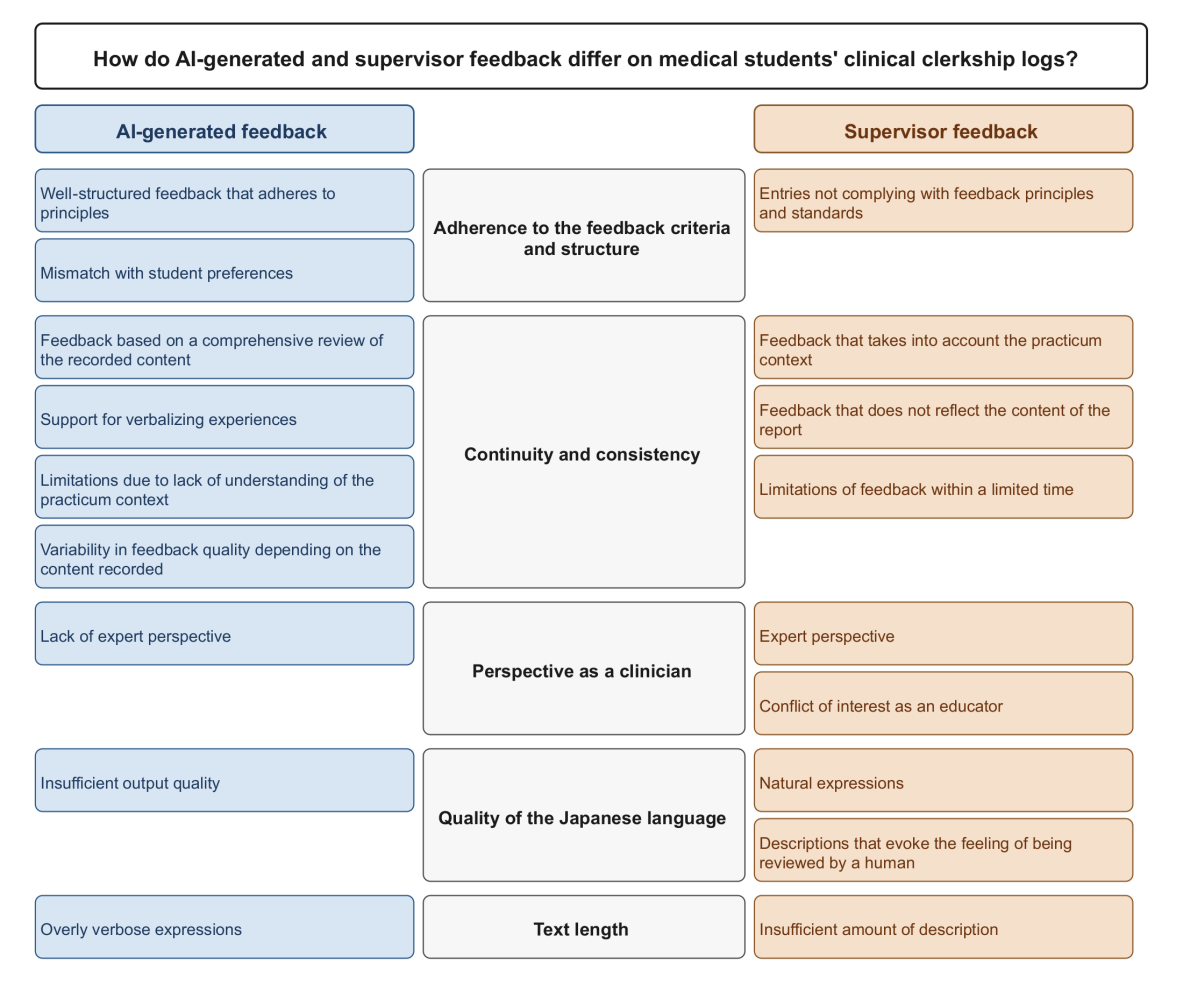
Thematic map of differences between AI-generated and supervisor feedback on medical students’ clinical clerkship logs. The research question is presented at the top, the 2 feedback modes are shown as contrasting poles, and each of the 5 themes is displayed with its AI and supervisor subthemes arranged on the left and right, respectively. Convergent mixed methods study, 161 clinical clerkship logs, Nagoya University, Japan, 2024. AI: artificial intelligence.

The qualitative analysis of the free-text comments provided by the evaluators revealed several themes regarding the differences between AI and supervisor feedback. Following the thematic-map guidance of Ahmed et al [50], the thematic map in Figure 5 visualizes the research question, the 5 themes, and their subthemes arranged by feedback provider.

Five themes were identified as differences between AI and supervisor feedback: “adherence to the feedback criteria and structure,” “continuity and consistency,” “perspective as a clinician,” “quality of the Japanese language,” and “text length.” Below, we describe the details of these themes. Citations consist of IDs with 3 parts. When the first part is “AI_,” it indicates comments about AI feedback, and when it is “SU_,” it indicates comments about supervisor feedback. The second part shows “_FA” for comments by faculty evaluators and “_ST” for comments by student evaluators. The number in the latter half represents the evaluator ID. The final part indicates the dataset ID.

#### Adherence to the Feedback Criteria and Structure

Across comments, AI-generated feedback was repeatedly described as tightly aligned with the rubric and highly structured. Evaluators highlighted concrete, example-driven suggestions, clear prioritization, and a predictable structure. This structure made the next steps “easy to put into practice,” but some judged it overly prescriptive or formulaic. By contrast, supervisor feedback often departed from the rubric’s structure: at times it omitted actionable “points for improvement,” offered too few details, or listed many items without prioritization. Several comments, however, framed these departures as deliberate, “living” feedback rooted in real clinical work rather than in formal criteria.

Illustrative comments included: “This is a well-written report that starts with positive feedback, is specific and constructive, and doesn’t have too many points” (AI_FA07_060), “It goes from positive to constructive ... priorities are also pointed out ... good feedback” (AI_FA07_063), and “It is very specific and detailed. It is written in the classic P-N-P format ...” (AI_FA09_086). On limits of the AI style, evaluators noted it could feel templated or prescriptive, eg, “sentences were ... not natural” (AI_ST18_078) and “it would be better if the suggestions for revision were not given in specific sentences” (AI_ST20_115). For supervisors, one comment praised specificity but worried that “there are many items, there is no priority, and it seems a little harsh” (SU_FA07_058), while many others pointed out missing action points: “There is no specific feedback ...” (SU_FA13_163) and “No evaluation was given at all. No feedback ... on areas for improvement” (SU_ST21_131). Several remarks captured the trade-off: “In terms of referring to the evaluation criteria, it is inferior ... but it is more in line with the content of the practical training and presents the next task” (SU_FA11_127) and “It’s not feedback that is tailored to a standard, but it feels like ‘living’ feedback that is likely to bring about behavioral change” (SU_FA09_088).

#### Continuity and Consistency

Across evaluators, continuity and consistency emerged as a defining difference in how AI and supervising physicians oriented their feedback. AI feedback typically exhibited strong internal coherence with the student’s clerkship log. It read closely, mirrored the structure of entries, and translated what was on the page into concrete, criterion-referenced suggestions. This text-anchored continuity helped AI surface specific, actionable edits to the log itself. At the same time, because AI focused only on the student clerkship log, it could miss or misinterpret the broader practicum context, weekend schedules, orientation days, unit norms, or feasibility in busy wards, and offered fewer comments about attitudes, behaviors, or clinical reasoning unfolding across the rotation. In contrast, supervising physicians showed continuity with the clinical experience. Their feedback often drew on shared encounters, observed behaviors, and ward-based realities, which gave comments a sense of lived coherence over time. However, that experiential continuity did not reliably connect back to the student’s written descriptions. Many supervisor comments were brief, global, or focused on the clinical day rather than the clerkship log, creating a misalignment.

This pattern was visible in how AI consistently tied remarks to what students had written: “the feedback is tailored to the clinical clerkship logs ... points that need to be improved ... are mentioned in detail, with specific examples” (AI_ST15_031) and “careful guidance in response to the lack of clinical clerkship logs ... specific suggestions for improvement” (AI_ST15_030). Evaluators repeatedly noted that AI “gave feedback after the log had been carefully examined ... [with] very specific” next steps (AI_ST21_117) and that its “comments are based on the content” (AI_FA05_034). Yet, this text-level consistency sometimes slipped when context mattered: AI “gives the impression that days without logs=days without practice” (AI_FA10_104) and tended to “encourage learning by improving how they write ... [with] a lack of clinical knowledge or perspective” (AI_ST21_121). Several evaluators wished for an overarching synthesis across the week or rotation: “no overall evaluation ... perhaps (AI was) a good micro-manager?” (AI_FA11_117) and “it would be even better if the feedback also took into account the overall outcome” (AI_FA10_109). By contrast, supervisors frequently referenced shared clinical situations and behaviors “useful because it gave supplementary information that wasn’t included in the medical student’s description” (SU_ST17_062); advice that was “practical and in line with real clinical practice” (SU_ST18_080); and impressions that only a clinician observing the student could offer, such as “he was going about things in a calm and collected way” (SU_ST23_157) and the feeling of “working together with medical students” (SU_FA04_008). However, this experiential continuity often bypassed the portfolio itself: “no mention of the content of the practical training or the learner’s own description” (SU_FA10_109), “they did not evaluate the logs that were written ... just a summary evaluation conclusion” (SU_FA11_119), and “feedback was based on the actual content of the practical training ... it would be better if there was more feedback on the learning logs” (SU_ST21_124). In some instances, identical or near-identical messages suggested time-pressured reuse rather than log-specific engagement (eg, “copied and pasted feedback” (SU_ST23_164)). Taken together, the data indicate that AI maintained continuity with what students wrote, whereas supervisors maintained continuity with what students did, each affording a different, and at times incomplete, alignment with the students’ log.

#### Perspective as a Clinician

Evaluators frequently contrasted the clinical lens. AI feedback was perceived as strong on structure, yet often thin on clinical reasoning, occasionally overlooking medical inaccuracies or proposing improvements not grounded in specialty-specific practice. Supervisor feedback, in turn, more often drew on clinical judgment, flagging errors, supplying pathophysiologic rationale, and pointing learners toward consequential problems, though some comments cautioned that supervisors can import their own priorities (eg, recruitment messages and niche emphases) or inadvertently signal an authority gradient.

Comments underscoring AI’s limits included: “did not point out the errors in the words ‘pulse irregularity’ and ‘electrocardiogram holder’” (AI_FA11_120), “specific examples ... are ideas that are not based on much medical expertise” (AI_ST22_143), “suggestions for improvement ... are not from the surgeon’s perspective” (AI_FA06_050), “fixated on relatively trivial points ... specific feedback lacks accurate knowledge” (AI_ST15_024), and “main points were comments on how to fill in the student logs ... lack of clinical feedback” (AI_ST21_123). By contrast, supervisor excerpts highlighted the expert stance: “points out errors in writing and correctly teaches medical knowledge” (SU_FA06_037), “I can get feedback on my knowledge, so it is useful for my studies” (SU_ST17_058), and advice “that would only be conveyed by a clinician, such as the long-term perspective of ‘going home’” (SU_ST23_150). Potential downsides were also noted: “content is more focused on recruiting students to the department” (SU_FA05_022) and concerns about prioritization, “few opportunities to do peripheral nerve examinations, but ... whether this is something that all medical students should learn” (SU_FA10_110), as well as an authority gradient, as exemplified by the following quote: “show the superiority and infallibility of the instructor ... makes learners feel an authority gradient” (SU_FA10_108).

#### Quality of the Japanese Language

Language quality emerged as a salient differentiator. AI feedback was often experienced as translation-like, occasionally inconsistent, and at times marred by formatting artifacts. Such phrasing could undermine credibility or supportive tone even when the substantive guidance was sound. Supervisor language, by comparison, was more often described as natural, human, and relationship-affirming.

Illustrative appraisals of AI language included: “Expressions such as ‘Great job!’ and ‘I will provide feedback.’ are English-Japanese translation-like ... unnatural as Japanese” (AI_ST23_149), “mysterious extra characters” (AI_ST18_083), “divided into paragraphs ... a bug or character corruption” (AI_FA08_083), and “very ChatGPT-like” (AI_ST17_054). Several noted formulaic tone: “the sandwich of good points ... is so formulaic that ... students might stop reading the feedback seriously” (AI_ST14_014), alongside word choice that “gives the impression of being a little unfriendly” (AI_FA10_108). In contrast, supervisor comments emphasized naturalness and human presence: “the advice is very good, accurate and natural” (SU_ST18_079) and “it feels like the individual is being looked at and taught” (SU_ST20_109).

#### Text Length

Across the dataset, AI feedback tended to be substantially longer, often enumerating many points and including concrete examples. Participants suggested that this length may help make expectations explicit and actionable, yet also noted that verbosity can hinder readability and reduce the salience of key priorities. By contrast, supervisor feedback was frequently very short—sometimes so brief that essential elements (eg, specific points for improvement) were missing. Still, several comments valued concision when it clearly conveyed the main point and respected time constraints.

Exemplars included the AI being “somewhat verbose” (AI_ST23_155) with “so many points ... it would be easier to understand if there was a summary” (AI_ST16_047). For supervisors, multiple comments judged entries too brief: “not possible to give feedback with just a few words” (SU_ST16_044), “just too short” (SU_FA05_020), “I don’t think a simple ‘Thank you for your hard work’ would be considered feedback” (SU_FA09_094), and “too simple and doesn’t amount to feedback” (SU_FA11_131). A counterexample highlighted that brevity can still work when targeted: “concise feedback that includes the necessary content ... motivates the student for future practice” (SU_ST15_034).

### Integration of Quantitative and Qualitative Results

#### Overview

JDA was used to integrate the results of the quantitative and qualitative analyses. The integration of quantitative and qualitative findings reveals 6 convergent patterns that illuminate the differences between AI-generated and supervisor-provided feedback. The results of the quantitative analysis are shown in the second column of Table 6, while the themes identified in the qualitative analysis are shown in the third column. The fourth column shows how the quantitative and qualitative results are related. Below, we explain the integrated insights obtained from each row.

**Table 6.** Joint display integrating quantitative and qualitative findings from a convergent mixed methods study comparing artificial intelligence (AI)–generated and supervisor feedback on clinical clerkship logs, Nagoya University, Japan, 2024.

Index	Quantitative results	Qualitative results	Type of integration
1	Feedback length difference (AI>supervisor)	Theme: “text length”	Quantitative results support qualitative findings Qualitative findings explain quantitative results
2	Comparison of rubric scores Feedback length	Theme: “adherence to the feedback criteria and structure” Theme: “text length”	Quantitative and qualitative results complement each other
3	Correlation between feedback length and rubric score (significant for supervisors and absent for AI)	Theme: “text length”	Combination of quantitative and qualitative results leads to new insights
4	Consistency of rubric scores	Theme: “adherence to the feedback criteria and structure”	Quantitative results support qualitative findings
5	Comparison of rubric scores	Theme: “continuity and consistency” Theme: “perspective as a clinician”	Qualitative results expand quantitative results
6	Identification of feedback source	Theme: “adherence to the feedback criteria and structure” Theme: “continuity and consistency” Theme: “perspective as a clinician” Theme: “quality of the Japanese language” Theme: “text length”	Qualitative findings explain quantitative results

#### Index 1: Text Length and Feedback Quality

The quantitative finding that AI feedback was significantly longer than supervisor feedback was substantiated by the qualitative theme of text length. Qualitative analysis revealed that AI feedback length enabled the provision of specific examples, contributing to clarity, while potentially resulting in verbose expressions with unclear priorities. Conversely, supervisor feedback brevity sometimes led to missing important elements but could be highly effective when concise and targeted. This integration demonstrates that qualitative findings explain the impact of length differences on feedback quality, which quantitative analysis alone could not reveal.

#### Index 2: Adherence to Rubric Criteria

Quantitatively, AI feedback scored significantly higher than supervisor feedback on the clear-direction and criteria-based items and showed a nonsignificant trend toward higher scores on the remaining items. These results were complemented by the qualitative theme of adherence to feedback criteria and structure and by the difference in text length. Qualitative analysis revealed that AI feedback followed the rubric with clear structure and provided actionable points, whereas supervisor feedback was often too brief and lacking some required elements. The significant difference in feedback length was also demonstrated quantitatively. This complementary integration explains how AI feedback aligns with the rubric’s structural criteria through both structural adherence and sufficient length.

#### Index 3: Length-Score Correlation Patterns

A particularly illuminating pattern emerged from combining quantitative correlation analysis with qualitative text length observations. Quantitative examination revealed that while there was no correlation between length and rubric scores in AI feedback, longer submissions received higher scores in supervisor feedback. Qualitative examination showed that AI feedback length enables specific examples to be provided, leading to clarity, while potentially resulting in verbose expressions with unclear priorities. Supervisor feedback brevity can lead to missing important elements but can be highly effective when concise and targeted. This suggests that AI more systematically covers the elements specified by the rubric, while supervisors excel at contextual prioritization. Combining these findings reveals that feedback requires sufficient length to include necessary items, while prioritization is also important, and longer is not necessarily better.

#### Index 4: Rubric-Score Consistency and Variability

The quantitative finding that AI feedback rubric scores were more consistent while supervisor feedback rubric scores were more variable was supported by qualitative observations regarding adherence to feedback criteria and structure. The qualitative analysis described AI feedback as closely following the rubric, leading to more uniform rubric scores, whereas supervisors often deviated from the rubric, resulting in variable scores. This integration demonstrates how structural consistency in AI feedback translates directly into measurable rubric-score consistency.

#### Index 5: Clinical Context and Perspective

The quantitative results regarding rubric scores were expanded by qualitative themes of continuity and consistency, as well as perspective as a clinician. Qualitative analysis revealed that AI feedback maintained continuity with written logs and provided specific text-level suggestions while missing broader clinical context. In contrast, supervisor feedback aligned with clinical experience but often failed to engage with specific portfolio content. These qualitative findings revealed aspects beyond those evaluated by the rubric, specifically demonstrating that supervisor feedback was grounded in clinical context and used expert perspectives, showing advantages not captured by the rubric scores.

#### Index 6: Distinguishability of Feedback Sources

The quantitative finding that all evaluators could completely distinguish between AI and supervisor feedback was explained by all 5 qualitative themes identified in the analysis. The themes of adherence to feedback criteria and structure, continuity and consistency, perspective as a clinician, quality of Japanese language, and text length each highlighted distinct differences between the 2 feedback sources. AI feedback followed the rubric with clear structure, maintained continuity with written logs, and was long with specific examples, while supervisor feedback excelled at contextual prioritization and provided feedback from a clinician perspective with natural, human expressions. These differences suggest why evaluators could distinguish feedback providers, though the analysis cannot determine whether evaluators considered these factors in combination or whether specific elements were decisive in their identification process.

## Discussion

### Principal Findings

This study compared AI-generated feedback with supervisor-provided feedback on medical student clinical clerkship logs through mixed methods analysis, integrating results using a JDA. AI feedback was significantly longer and demonstrated greater adherence to rubric-based criteria, particularly in providing criteria-based guidance and clear directions for improvement. While supervisor feedback showed greater variability in rubric scores, with length correlating positively with those scores, AI feedback maintained consistent rubric scores regardless of length, suggesting systematic coverage of the structural elements captured by the rubric. Qualitative analysis revealed complementary strengths: AI provided text-anchored feedback with structured, comprehensive coverage of student log content, while supervisors offered experience-based insights grounded in clinical context and professional expertise. Despite AI’s structural advantages, supervisor feedback was valued for its clinical perspective and natural language use. All evaluators could reliably distinguish between the 2 feedback sources, reflecting substantial differences in approach, structure, and content focus.

### Comparison With the Literature

Our findings align with and extend previous research on AI-generated feedback in several key areas. Regarding feedback length, prior studies have reported various findings [20,23-25]. Our study demonstrated that AI feedback was significantly longer than supervisor feedback, which is consistent with some previous findings [20,23,24]. In our research, the supervisors providing feedback were working in busy clinical environments, which likely contributed to their tendency to provide shorter feedback. Furthermore, our results showed no correlation between AI feedback length and rubric scores, while supervisor feedback demonstrated a positive correlation between length and rubric scores. Some reports indicate that feedback length does not contribute to quality improvement beyond a certain threshold [51]. Considering that AI feedback length was sufficiently longer compared to supervisor feedback, our findings are consistent with these previous studies. Prior research has pointed out that busy physicians tend to provide short feedback, often lacking important elements such as specific examples and future action guidelines [28,52]. Our findings suggest that AI-generated feedback may be able to complement elements that tend to be missing in supervisors’ brief feedback.

Regarding consistency in feedback quality, previous research has shown mixed results about whether AI or human feedback demonstrates greater consistency [25,29-32]. Our study found that AI feedback exhibited higher consistency in rubric scores compared to supervisor feedback. It is important to distinguish AI’s adherence to rubric criteria, which follows from embedding those criteria in the prompt, from the consistency of its output across different inputs. The latter is a structural consequence of using the same model and prompt configuration for every log entry, which constrains the range of possible outputs. This consistency is therefore best understood as a design feature of AI feedback generation rather than an inherently desirable educational property, as effective feedback may sometimes require selective emphasis tailored to individual learner needs. In contrast, the greater variability observed in supervisor feedback likely reflects selective prioritization and clinical judgment: supervisors chose which aspects to emphasize based on their professional assessment of each student’s needs, resulting in variable but contextually responsive feedback. Previous research has identified multiple factors that influence feedback quality variability, including feedback length [29], the subject of feedback [30], and whether supervisors have received feedback training [53]. Our findings suggest that in busy clinical environments, AI could provide structurally stable feedback that complements the individualized, experience-driven feedback of supervisors.

The distinction between text-anchored versus experience-based feedback represents a novel contribution of our study. Most prior comparisons of AI and human feedback have been conducted in classroom or simulation settings [22,24,25,54], where the knowledge base is relatively well-defined and the feedback task is circumscribed. Our study extends this comparison to an authentic clinical clerkship environment, where several contextual features distinguish the feedback task. Supervisors work under substantial time pressure and provide written feedback as a complement to ongoing face-to-face clinical teaching, resulting in brief comments that prioritize what each supervisor judges most important for a given student. Clinical reasoning, situational awareness, and tacit professional knowledge, which are central to clerkship learning [55], are difficult to articulate in written form [56] and largely inaccessible to AI systems that operate solely on the written log text. Previous research has noted that AI feedback tends to provide comprehensive feedback closely tied to the target text [24,25], while human experts in specialized fields provide context-informed feedback that is perceived to be of higher quality [22,54]. Our findings confirm and deepen this pattern: AI feedback was more likely to provide text-consistent feedback aligned with students’ written clerkship logs, while supervisor feedback drew on clinical experience, the broader educational relationship, and professional judgment that extends well beyond what is documented in the log. These contextual features of clinical clerkships amplify the qualitative differences between AI and supervisor feedback compared to what has been observed in classroom settings and underscore the importance of studying AI feedback in authentic clinical environments where the complementary nature of each feedback source is most evident.

Regarding the ability to distinguish between AI and human feedback, previous studies have reported inconsistent findings [33-35]. In our study, all evaluators correctly identified the source of feedback with 100% accuracy, despite blinding procedures that included randomized presentation order, removal of source labels, and placement of the identification question after all rubric scoring and free-text comments had been completed. The differences we observed in text length, feedback structure, and clinical perspective in the busy clinical environment likely served as decisive factors for identification, consistent with all 5 qualitative themes that highlighted distinct textual characteristics of each feedback type.

This perfect detection has implications that warrant discussion along 2 distinct lines. The first concerns the evaluation itself: because evaluators recognized the feedback source with complete accuracy, they may have formed implicit judgments about the source during rubric scoring even though they were not explicitly informed until afterward, and the possibility that such recognition shaped ratings cannot be ruled out. Prior research has demonstrated that knowledge or perception of the feedback provider can influence how feedback is received and evaluated [45,46], and a recent controlled-content experiment further showed that source attribution altered learner engagement by a large margin (Cohen *d*=0.88‐1.56) even when the feedback content was held identical [57]. In our study, however, the identification question was administered only after all rubric scoring and free-text comments had been completed, so any such influence would have arisen from the textual characteristics of the feedback rather than from explicit labels, unlike an unblinded evaluation in which the source is known a priori.

The perfect detection also constitutes a substantive finding in its own right. The 5 qualitative themes identified in our analysis (adherence to feedback criteria and structure, continuity and consistency, perspective as a clinician, quality of the Japanese language, and text length) collectively describe the lexical, structural, and contextual features that made AI feedback identifiable in this Japanese-language clinical context, with language-level cues such as translation-like phrasing, formatting artifacts, and a formulaic register being particularly salient. At the level of interpretation, these differences suggest that AI and supervisor feedback differ not only in structural properties but in the epistemic voice they carry: supervisor feedback is grounded in lived clinical encounter, whereas AI feedback operates on textual pattern. Similar tensions around authenticity and voice have been raised in recent medical education writing on AI-assisted reflective work [58]. This voice differential can be read positively as a form of source transparency, since learners and educators are unlikely to mistake one source for the other, but also as an authenticity gap that may affect trust and uptake when AI feedback is delivered to learners. Newer LLMs continue to improve in generating natural, human-like text, and future iterations may substantially reduce the detectability of AI-generated feedback, potentially altering both evaluator perceptions and the dynamics of blinded evaluation designs.

An important consideration for interpreting our findings is the linguistic and cultural context in which this study was conducted. All feedback was generated and evaluated in Japanese, and LLM performance has been shown to vary substantially across languages. Strasser et al [59] demonstrated that LLM accuracy on identical medical examination questions differed significantly by language (64%‐87%), with English prompts generally yielding higher performance, although advanced models showed comparable results with language-matched prompts in some languages. Harigai et al [60] found that GPT-4 achieved significantly higher accuracy on English-translated radiology questions (median 89 points) than on the original Japanese versions (median 70 points), attributing this gap to the predominance of English in training data and to translation challenges specific to Japanese, including logographic script ambiguity and structural differences. These findings suggest that the quality of AI-generated feedback in our study may be partly shaped by GPT-4o’s language-specific capabilities in Japanese, and that the same prompt and model configuration could produce qualitatively different feedback in other languages. Furthermore, the cultural context of Japanese medical education, where deference to senior clinicians is strongly emphasized [61], may have influenced evaluators’ perceptions of and receptivity to the 2 feedback sources. Rather than framing these contextual factors solely as limitations, we note that they underscore the importance of conducting AI feedback research across diverse linguistic and cultural settings, as both AI performance and user reception are likely to be context-dependent.

### Implications of Findings

These findings have important implications for the integration of AI-generated feedback in clinical education. Our results suggest that AI-generated and supervisor-provided feedback each possess distinct strengths and limitations and should be leveraged in a complementary manner rather than as replacements for one another. For instance, AI could provide structured, criterion-based feedback alongside supervisor feedback, allowing medical students to benefit from both perspectives. However, such direct provision of AI feedback requires careful consideration of potential risks, including student overreliance on AI recommendations [62] or conversely, dismissal of AI-generated insights [20]. Our finding that evaluators recognized feedback source with perfect accuracy suggests that learners in real-world settings would plausibly do the same, and evidence from controlled-content experiments indicates that source attribution can substantially alter learner engagement when feedback is delivered directly (Cohen *d*=0.88‐1.56) [57]. The rubric-score advantages we observed should therefore not be assumed to translate directly into comparable learner uptake or educational impact when AI feedback is delivered to students who can identify its source.

Various hybrid approaches merit exploration, including AI-drafted feedback refined by educators, role-based distribution where AI and supervisors focus on different aspects of student work, or AI enhancement of educator-authored feedback [34,63]. However, empirical research on these hybrid models remains limited, particularly in authentic clinical settings.

Underlying both direct delivery and hybrid configurations is the prompt itself, an object of pedagogical design that shapes what the AI produces in any deployment model. In our case, embedding the rubric criteria directly in the prompt operationalized a particular conception of feedback quality in AI generation. This is analogous to constructive alignment in instructional design [64], where intended outcomes are aligned with the activities producing them. Recent applied guidance in health professions education has similarly recommended grounding generative AI prompts in institutional pedagogical and assessment frameworks [65]. The rubric-score consistency we observed for AI feedback is therefore best read not as an inherent property of GPT-4o but as an outcome of this prompt configuration, consistent with experimental evidence that detailed prompts combined with low temperature settings yield near-perfect interreplicate consistency in LLM rubric grading [66]. The same design lever is available to other educators and institutions seeking to align AI feedback with their own assessment frameworks.

Future research should examine how learners respond to and use AI-generated feedback and investigate the effectiveness of hybrid approaches that combine AI and supervisor feedback. Such studies will be essential for determining optimal implementation strategies for AI feedback systems in clinical clerkship environments, ensuring that technological capabilities enhance rather than disrupt the educational relationships and learning processes that are fundamental to clinical training.

### Limitations

Several limitations should be acknowledged in this study.

First, as this research was conducted at a single institution in Japan, the generalizability of our findings to other institutions or cultural contexts needs to be confirmed. The study was conducted entirely in Japanese, and GPT-4o’s performance in Japanese may differ from its performance in English, potentially affecting the naturalness and quality of AI-generated feedback. Additionally, the cultural context of Japanese medical education, where deference to senior clinicians is strongly emphasized [61], may have contributed to higher regard for supervisor feedback and influenced evaluators’ perceptions of the 2 feedback sources. The specific characteristics of NU’s clinical clerkship program may further limit representativeness.

Second, while our evaluators included both faculty and students, their individual backgrounds and experiences may have influenced their feedback evaluations. The relatively small number of evaluators (10 faculty and 10 students) may also limit the robustness of our findings.

Third, all evaluators correctly identified the source of every feedback entry despite blinding procedures (randomized presentation order and removal of source labels). Although the identification question was administered only after all rubric scoring and free-text comments had been completed, the perfect detection accuracy indicates that the textual characteristics of the 2 feedback types were highly salient and that evaluators may have formed implicit judgments about the source during the evaluation process. Because all evaluators correctly identified every item, perceived source and actual source are identical, making it logically impossible to stratify scores by perceived source to estimate the magnitude of any expectancy bias. Readers should therefore consider the possibility that implicit source recognition may have influenced rubric ratings, particularly for items on which AI scored highest.

Fourth, this study used a specific AI model (GPT-4o) for feedback generation, and results may differ with other AI models or future iterations of the technology. The prompt engineering process, while systematic, was tailored to our specific context and may not be optimal for other settings.

Fifth, the evaluation rubric used to assess feedback quality was originally developed for written academic feedback [18] and emphasizes structural dimensions such as criteria-based guidance, clarity of directions, and text alignment. Clinically meaningful attributes of feedback, such as credibility, diagnostic reasoning, situational awareness, and professional judgment, are not directly captured by this instrument. The rubric score differences between AI and supervisor feedback therefore reflect adherence to these specific structural dimensions rather than overall educational quality. The qualitative findings, particularly the themes of perspective as a clinician and continuity with practice, provide complementary evidence of the clinical value that supervisors contribute and that the rubric does not score.

Sixth, the absence of a prompt sensitivity analysis limits the interpretability of our findings. It remains unclear whether the observed rubric score advantages for AI feedback reflect inherent properties of GPT-4o or are contingent on this specific, carefully engineered prompt. Alternative prompts, whether shorter, less structured, or more clinically oriented, might produce qualitatively different comparative outcomes. Conducting a systematic sensitivity analysis was beyond the scope of this study, as the prompt was developed iteratively through a pilot testing process, and varying it systematically would constitute a separate study design. This remains an important direction for future research.

Seventh, each dataset was evaluated by only 1 faculty member and 1 student, rather than by multiple independent raters. Interrater agreement was fair to moderate for supervisor feedback but poor for AI feedback, indicating that individual evaluator perspectives influenced ratings, particularly when assessing AI-generated output. Although the large number of datasets (n=161) provides stability at the aggregate level, the precision of scores for individual datasets is limited.

Eighth, our study evaluated the structural quality of feedback as assessed by third-party evaluators using the rubric, but did not examine whether AI-generated or supervisor-provided feedback, when delivered to students, elicited different learning behaviors, revisions, engagement, or clinical improvement. Because our design measured judgments by evaluators rather than responses by learner recipients, source-attribution effects that may arise when identifiable AI feedback is delivered directly to students [57] fall outside the scope of our data. The relationship between perceived feedback quality, source attribution, and actual educational outcomes remains an important area for future investigation.

### Conclusions

This study extends the comparison of AI-generated and supervisor feedback from classroom and simulation settings to an authentic clinical clerkship environment, where time pressure, complex professional expertise, and ongoing educational relationships shape the feedback process. Through integrated mixed methods analysis, a key distinction emerged between text-anchored AI feedback, which systematically addresses written log content in alignment with rubric criteria, and experience-based supervisor feedback, which draws on clinical observation and professional judgment. Using GPT-4o with a rubric-embedded prompt, AI consistently delivered structured feedback addressing gaps that arise when time-pressured supervisors provide brief comments, while supervisors contributed clinically grounded insights that AI cannot replicate. These complementary strengths suggest that AI feedback should supplement rather than replace supervisor feedback in clinical education. Future research should investigate hybrid models that leverage each type’s advantages; examine whether these patterns generalize to other AI models, languages, and institutional contexts; and evaluate the impact of AI-assisted feedback on student learning outcomes.

## Supplementary material

10.2196/90064 Multimedia Appendix 1 The feedback generation prompt for artificial intelligence used in this study and its development process.

10.2196/90064 Multimedia Appendix 2 The feedback evaluation rubric used in this study.

10.2196/90064 Multimedia Appendix 3 The translation process into Japanese for the feedback evaluation rubric used in this study.

10.2196/90064 Multimedia Appendix 4 Distribution of rubric score differences between artificial intelligence–generated and supervisor feedback by rubric item and assessor type.

10.2196/90064 Multimedia Appendix 5 The full paired *t* test results comparing artificial intelligence and supervisor feedback scores on the Steiss et al 5-item analytic rubric, including mean differences and uncorrected *P* values omitted from Table 2 for readability.

## Abbreviations

AI: artificial intelligence
API: application programming interface
JDA: joint display analysis
LLM: large language model
NU: Nagoya University
OR: odds ratio
