# Supplementary material for: AI-Generated Versus Human Supervisor Feedback on Medical Students’ Clinical Clerkship Logs: Cross-Sectional Convergent Mixed Methods Study
Source: JMIR Med Educ. 2026 Jun 16;12:e90064. doi: 10.2196/90064 (PMC13271589; doi:10.2196/90064)
Supplement: Multimedia Appendix 2 [file mededu-v12-e90064-s002.pdf]

Feedback Evaluation Form

One of feedback_1 or feedback_2 is actual feedback from a supervisor, and the other is automatically generated by AI. It is randomly assigned which one is generated by AI.

Medical students are instructed to record the following as "criteria" during clinical practice:
- Record daily
- Describe what they experienced in detail and specifically
- Describe what they learned in detail and specifically
- Describe future goals in detail and specifically

Additionally, the following are instructed as "criteria" for behavior during clinical practice:
- Be punctual and notify in case of tardiness or absence
- Pay attention to attire and grooming, and wear a name tag
- Communicate appropriately with patients and the medical team
- Learn actively and independently (actively interact with patients, actively participate in conferences, complete assignments)

Based on the above, please evaluate feedback_1 and feedback_2, which are feedback on the student's practice records.

id*

Automatically filled. No action needed.

Criteria-based*

Score Category: Criteria-based feedback should explicitly reference criteria for student log.

	5: Consistently explicitly references criteria of student log ; minimal to no feedback is generic.	4: Most feedback references criteria; some feedback is generic.	3: Half of the feedback explicitly references criteria and half is generic.	2: Most feedback is generic; one piece somewhat references criteria.	1: Does not explicitly reference criteria; all feedback is generic.
feedback_1	○	○	○	○	○
feedback_2	○	○	○	○	○

Clear directions for improvement*

Score Category: Feedback should mark what a writer has done well and be specific about where and how they can improve.

	5: Consistently gives clear directions for improvement; offers specific examples; all feedback is clear.	4: Mostly offers usable feedback; some is less clear.	3: Even mix of specific and vague suggestions.	2: Minimal references to specific student writing or lacks actionable steps.	1: Does not reference specific student writing and does not give concrete steps.
feedback_1	○	○	○	○	○
feedback_2	○	○	○	○	○

Accurate*

Score Category: Feedback must be accurate for cognitive and affective reasons.

	5: All feedback is accurate.	4: Most feedback is accurate; one piece is somewhat inaccurate.	3: Some feedback is accurate; some clearly inaccurate.	2: Feedback is mostly inaccurate.	1: Feedback is inaccurate, irrelevant to student log-keeping .
feedback_1	○	○	○	○	○
feedback_2	○	○	○	○	○

Prioritization of essential features*

Score Category: Feedback should respond to appropriate elements of daily logs and mention the next step in development.

	5: All feedback focuses on the most appropriate priority to work on.	4: Most feedback is attainable and reasonable; prioritizes one of the most appropriate things.	3: Even mix of prioritizing essential and nonessential features.	2: Most feedback is unattainable, nonessential, or inappropriate.	1: Feedback is too difficult or does not mention any essential features.
feedback_1	○	○	○	○	○
feedback_2	○	○	○	○	○

Supportive tone*

Score Category: Quality of feedback ranges from unsupportive and directive to affirming and supportive.

	5: Consistently affirming; uses suggestive and respectful language.	4: Most feedback uses suggestive/supportive language, some does not.	3: Even mix of suggestive/directive language.	2: Most language is directive or lacks any positive affirmation.	1: No positive comments and no suggestive, respectful language.
feedback_1	○	○	○	○	○
feedback_2	○	○	○	○	○

Which one do you think was generated by AI?*

One of feedback_1 or feedback_2 is actual feedback from a supervisor, and the other is automatically generated by AI. Please tell us which one you think was generated by AI.

○ feedback_1 was generated by AI

○ feedback_2 was generated by AI

About feedback_1*

Considering using feedback to contribute to the learning of medical students, please tell us your impressions, good points, and points for improvement of feedback_1.

About feedback_2*

Considering using feedback to contribute to the learning of medical students, please tell us your impressions, good points, and points for improvement of feedback_2.

RESET SEND
