# Supplementary material for: AI-Generated Versus Human Supervisor Feedback on Medical Students’ Clinical Clerkship Logs: Cross-Sectional Convergent Mixed Methods Study
Source: JMIR Med Educ. 2026 Jun 16;12:e90064. doi: 10.2196/90064 (PMC13271589; doi:10.2196/90064)
Supplement: Multimedia Appendix 3 [file mededu-v12-e90064-s003.docx]

# Translation Process

The translation process was conducted based on the TRAPD model [@Walde2023-vi]. This model is designed for the translation of evaluation forms, aiming to perform accurate translations while adapting to linguistic characteristics and cultural contexts that are difficult to address in traditional translation processes, and to maintain the intent of the evaluation forms. The model follows these five steps:

1. **T**ranslation: Two independent translators perform the translation.
2. **R**eview: A team including the translators and reviewers reviews the translation.
3. **A**djucation: A coordinator resolves any remaining inconsistencies after the review.
4. **P**retesting: The translated evaluation form is presented to subjects, feedback is obtained, and revisions are made.
5. **D**ocumentation: The selection of the translation process is documented.

## 0. Minimum Necessary Arrangements

The original feedback quality evaluation form was taken from [@Steiss2024-ra]. This questionnaire is an English evaluation form designed to assess the quality of written feedback. The form included parts that were specific to the context of the paper. Through deliberation by the research team (Takeshi, Sanne, Jeroen, Sylvia), the form was arranged to suit the current study without altering its general intent. Specifically, “quality source-based argument writing in history (SBAW)” was context-specific to the referenced paper, so it was modified to the more general term “criteria for student log.” Additionally, “writing” was adjusted to “log” or “student log” to fit the context of this study.

## 1. **T**ranslation

The translation was carried out by two native Japanese speakers. Takeshi Kondo and Hiroshi Nishigori, who are both native Japanese speakers, clinical practitioners, and medical education researchers, performed the translation. Takeshi holds a master’s degree in medical education, and Hiroshi has a PhD in medical education, both in English, and both have experience writing papers in English. The two independently translated the English questionnaire. While translating, they aimed to understand the original intent of the text and to ensure that the translation maintained that intent, adapting it to the cultural and medical education context in Japan.

## 2. **R**eview

In addition to the two translators, Dr. Noriki Kamihiro, a clinical practitioner, medical education researcher, and experienced writer of English papers, participated as a reviewer. The review session was conducted through a 1.5-hour synchronous online meeting and follow-up email exchanges. Takeshi’s translation adhered relatively closely to the original text, while Hiroshi’s translation was more adapted to the Japanese culture and the medical education context. After confirming the consistency, comprehensibility, grammatical accuracy, and retention of the original intent among the three participants, Hiroshi’s translation was judged to better reflect the original intent. Therefore, revisions were made to maintain overall consistency, using Hiroshi’s translation as the base and referring to Takeshi’s translation. Any unresolved parts during the review were left as comments and addressed during the adjudication process.

## 3. **A**djudication

In addition to the review members, Mr. Oliver Stanyon, a professional translator whose native language is English and who regularly proofreads medical education research papers, participated as an adjudicator. The adjudicator resolved the discrepancies that remained after the review by creating revision proposals and comments based on the discussions between the translators and the reviewer. Considering the nuances of the words used in the original text, the adjudicator made revisions to the Japanese expressions. The adjudicator also made some adjustments to the translated text to maintain overall consistency. After incorporating the adjudicator’s revisions, the two translators and the reviewer made further adjustments, considering the nuances and consistency of the Japanese language, and submitted the sheet to the adjudicator. The adjudicator agreed with the revised sections, completing the adjudication step.

## 4. **P**retesting

The translated evaluation form is intended for use by clinical educators and medical students. Therefore, pretesting was conducted with one clinical educator and one medical student. Dr. Naoki Yamada, a clinical educator and researcher, who also practices in clinical settings, served as the pretester for educators. Mr. Dai Iwata, a medical student in his final year who had completed clinical clerkship, served as the pretester for students. The pretesting was conducted using feedback on clinical clerkship records left by students at Nagoya University in 2024, as well as feedback generated by ChatGPT. As a result of pretesting, Dr. Yamada did not identify any issues with comprehensibility, while Dai pointed out that the criteria for evaluating student logs and the parts that emphasized the need for accuracy due to cognitive and emotional reasons were difficult to understand. However, Takeshi, Hiroshi, Noriki, and Oliver discussed and decided not to modify these parts to preserve the intent of the original. This concluded the pretesting, finalizing the translated questionnaire.

## 5. **D**ocumentation

The translation process was documented at every step, including comments from translators, reviewers, adjudicators, and pretesters. The documented translation process was shared within the research team along with the translated questionnaire.

## References

Steiss, Jacob, Tamara Tate, Steve Graham, Jazmin Cruz, Michael Hebert, Jiali Wang, Youngsun Moon, Waverly Tseng, Mark Warschauer, and Carol Booth Olson. 2024. “Comparing the Quality of Human and ChatGPT Feedback of Students’ Writing.” *Learning and Instruction* 91 (June): 101894. <https://doi.org/10.1016/j.learninstruc.2024.101894>.

Walde, Peggy, and Birgit Angela Völlm. 2023. “The TRAPD Approach as a Method for Questionnaire Translation.” *Front. Psychiatry* 14 (June): 1199989. <https://doi.org/10.3389/fpsyt.2023.1199989>.
