# Supplementary material for: AI-Generated Versus Human Supervisor Feedback on Medical Students’ Clinical Clerkship Logs: Cross-Sectional Convergent Mixed Methods Study
Source: JMIR Med Educ. 2026 Jun 16;12:e90064. doi: 10.2196/90064 (PMC13271589; doi:10.2196/90064)
Supplement: Multimedia Appendix 4 [file mededu-v12-e90064-s004.docx]

Convergent mixed-methods study, Nagoya University, Japan, 2024. For each of the 161 clinical clerkship logs, the score difference was computed as the AI score minus the supervisor score for each rubric item, separately for the faculty assessor and the student assessor. Cells show the number of logs at each difference value (d), ranging from -4 to 4; positive values indicate a higher AI score. n is the number of logs; Mean diff and SD summarize the difference distribution.

| Rubric item | Assessor | d=-4 | d=-3 | d=-2 | d=-1 | d=0 | d=1 | d=2 | d=3 | d=4 | n | Mean diff | SD |
| --- | --- | --- | --- | --- | --- | --- | --- | --- | --- | --- | --- | --- | --- |
| Criteria-based | Faculty | 1 | 0 | 3 | 3 | 32 | 24 | 33 | 34 | 31 | 161 | 1.88 | 1.62 |
| Criteria-based | Student | 1 | 5 | 7 | 9 | 34 | 22 | 25 | 17 | 41 | 161 | 1.52 | 2.03 |
| Clear direction | Faculty | 0 | 2 | 4 | 10 | 43 | 22 | 23 | 34 | 23 | 161 | 1.48 | 1.73 |
| Clear direction | Student | 2 | 7 | 3 | 11 | 44 | 22 | 16 | 24 | 32 | 161 | 1.29 | 2.04 |
| Accurate | Faculty | 3 | 0 | 15 | 27 | 55 | 20 | 17 | 15 | 9 | 161 | 0.41 | 1.72 |
| Accurate | Student | 2 | 2 | 11 | 25 | 71 | 20 | 7 | 10 | 13 | 161 | 0.34 | 1.67 |
| Prioritization | Faculty | 0 | 4 | 10 | 30 | 44 | 28 | 14 | 22 | 9 | 161 | 0.60 | 1.72 |
| Prioritization | Student | 2 | 7 | 11 | 17 | 51 | 23 | 11 | 11 | 28 | 161 | 0.76 | 2.06 |
| Supportive | Faculty | 0 | 0 | 2 | 20 | 101 | 18 | 10 | 7 | 3 | 161 | 0.29 | 1.07 |
| Supportive | Student | 4 | 4 | 9 | 11 | 96 | 14 | 7 | 8 | 8 | 161 | 0.17 | 1.55 |

*Abbreviation: SD, standard deviation.*
