# Supplementary material for: AI-Generated Versus Human Supervisor Feedback on Medical Students’ Clinical Clerkship Logs: Cross-Sectional Convergent Mixed Methods Study
Source: JMIR Med Educ. 2026 Jun 16;12:e90064. doi: 10.2196/90064 (PMC13271589; doi:10.2196/90064)
Supplement: Multimedia Appendix 5 [file mededu-v12-e90064-s005.docx]

| **Item** | **AI Mean Score (95% CI)** | **Supervisor Mean Score (95% CI)** | **Mean Difference (95% CI)** | **Cohen d (95% CI)** | **P value** | **Corrected P value** |
| --- | --- | --- | --- | --- | --- | --- |
| (A) Criteria Based | 4.43 (4.34-4.52) | 2.73 (2.56-2.9) | 1.7 (1.5- 1.9) | 0.93 (0.79 to 1.06) | <0.001 | <0.001 |
| (B) Clear Direction | 4.36 (4.26-4.46) | 2.98 (2.81-3.14) | 1.39 (1.18- 1.59) | 0.73 (0.61 to 0.86) | <0.001 | <0.001 |
| (C) Accurate | 4.18 (4.07-4.28) | 3.8 (3.64-3.96) | 0.38 (0.19- 0.56) | 0.22 (0.11 to 0.33) | <0.001 | <0.001 |
| (D) Prioritization | 4.05 (3.94-4.16) | 3.37 (3.2-3.54) | 0.68 (0.47- 0.88) | 0.36 (0.24 to 0.47) | <0.001 | <0.001 |
| (E) Supportive | 4.56 (4.47-4.65) | 4.33 (4.21-4.45) | 0.23 (0.08- 0.38) | 0.17 (0.06 to 0.28) | 0.002 | 0.011 |
